# *Terminalia catappa* Exerts Antimetastatic Effects on Hepatocellular Carcinoma through Transcriptional Inhibition of Matrix Metalloproteinase-9 by Modulating NF-**κ**B and AP-1 Activity

**DOI:** 10.1155/2012/595292

**Published:** 2012-11-08

**Authors:** Chao-Bin Yeh, Ming-Ju Hsieh, Yih-Shou Hsieh, Ming-Hsien Chien, Pen-Yuan Lin, Hui-Ling Chiou, Shun-Fa Yang

**Affiliations:** ^1^School of Medicine, Chung Shan Medical University, 110 Chien-Kuo N. Road, Section 1, Taichung 402, Taiwan; ^2^Department of Emergency Medicine, Chung Shan Medical University, 110 Chien-Kuo N. Road, Section 1, Taichung 402, Taiwan; ^3^Department of Emergency Medicine, Chung Shan Medical University Hospital, 110 Chien-Kuo N. Road, Section 1, Taichung 402, Taiwan; ^4^School of Medical Laboratory and Biotechnology, Chung Shan Medical University, 110 Chien-Kuo N. Road, Section 1, Taichung 402, Taiwan; ^5^Institute of Biochemistry and Biotechnology, Chung Shan Medical University, 110 Chien-Kuo N. Road, Section 1, Taichung 402, Taiwan; ^6^Wan Fang Hospital, Taipei Medical University, Taipei, Taiwan; ^7^Graduate Institute of Clinical Medicine, College of Medicine, Taipei Medical University, Taipei, Taiwan; ^8^School of Pharmacy, Taipei Medical University, Taipei, Taiwan; ^9^Institute of Medicine, Chung Shan Medical University, 110 Chien-Kuo N. Road, Section 1, Taichung 402, Taiwan; ^10^Department of Medical Research, Chung Shan Medical University Hospital, 110 Chien-Kuo N. Road, Section 1, Taichung 402, Taiwan

## Abstract

High mortality and morbidity rates for hepatocellular carcinoma (HCC) in Taiwan primarily result from uncontrolled tumor metastasis. Previous studies have identified that *Terminalia catappa* leaf extracts (TCE) exert hepatoprotective, antioxidative, antiinflammatory, anticancer, and antimetastatic activities. However, the effects of TCE on HCC and the underlying molecular mechanisms of its activities have yet to be fully elucidated. The present study's findings demonstrate that TCE concentration dependently inhibits human HCC migration/invasion. Zymographic and western blot analyses revealed that TCE inhibited the activities and expression of matrix metalloproteinase-9 (MMP-9). Assessment of mRNA levels, using reverse transcriptase polymerase chain reaction (PCR) and real-time PCR, and promoter assays confirmed the inhibitory effects of TCE on MMP-9 expression in HCC cells. The inhibitory effects of TCE on MMP-9 proceeded by upregulating tissue inhibitor of metalloproteinase-1 (TIMP-1), as well as suppressing nuclear translocation and DNA binding activity of nuclear factor-kappa B (NF-**κ**B) and activating protein-1 (AP-1) on the MMP-9 promoter in Huh7 cells. In conclusion, TCE inhibits MMP-9 expression and HCC cell metastasis and, thus, has potential use as a chemopreventive agent. Its inhibitory effects are associated with downregulation of the binding activities of the transcription factors NF-**κ**B and AP-1.

## 1. Introduction

Hepatocellular carcinoma (HCC) is a common malignant neoplasm and major cause of cancer-related deaths in Asian countries. A high mortality rate for HCC in Taiwan is principally caused by uncontrolled tumor invasion and metastasis [1]. Cancer cell metastasis involves multiple processes and various cytophysiological changes, including changes in the adhesive capability between cells and the extracellular matrix (ECM). This involves proteolytic degradation and damaged intracellular interaction. Degradation of the ECM by cancer cells occurs through proteases, such as serine proteinase and the matrix metalloproteinases (MMPs). This leads to the separation of the intercellular matrix to promote the mobility of cancer cells, eventually leading to metastasis [2]. Matrix metalloproteinase-9 (MMP-9 or gelatinase B, 92 kDa) is the proteases most significantly involved in the degradation of the basement membrane and, thus, in tumor invasion and metastasis. A number of pathological states, including cancer, inflammation, and vascular diseases, are associated with increased proteinase activities [3, 4]. The expression of MMPs is regulated by various factors, such as growth factors, cytokines, and proteinase inhibitors. The endogenous tissue inhibitors of metalloproteinases (TIMPs) are the specific inhibitors of MMPs. Imbalance between the MMPs and TIMPs might, therefore, contribute to degradation or deposition of the ECM [5, 6]. Inhibition of MMP-mediated migration or invasion could thus provide a means of preventing cancer metastasis.

*Terminalia* is a genus of combretaceous plants widely distributed in tropical and subtropical regions. In several Asian countries, physicians have used the leaves, bark, and fruit of *Terminalia catappa* to treat dermatitis and pyresis. *Terminalia catappa* leaf extracts (TCE) contain flavonoids and hydrolysable tannins, which have preventive effects on hepatoma and reduce hepatotoxicity. The antioxidative, hepatoprotective, anti-inflammatory, and carcinogenesis-preventing effects of TCE could potentially provide benefits to human health [7–9]. The group's previous study showed that TCE can inhibit lung cancer metastasis *in vivo* and *in vitro* [9]. However, the effects of TCE on HCC invasion and metastasis, and the underlying mechanisms of the antimetastatic effects, have yet to be evaluated. The present study, therefore, investigated the potential inhibitory effects of TCE on hepatocellular carcinoma (Huh7 cell) invasiveness, and the possible mechanisms of TCE-induced antimetastatic effects.

## 2. Materials and Methods

### 2.1. Preparation of Terminalia catappa Leaves Extracts (TCE)

*Terminalia catappa* leaves were purchased from local herb stores in Taichung and the TCE were prepared by an initial condensation followed by lyophilization as described previously [9]. Briefly, 100 g of air-dried leaves were boiled at 70°C for 24 h with 500 mL of 50% ethanol. The extraction procedure was repeated twice. Then, solvent was removed from the combined extract with a vacuum rotary evaporator. The filtrate was then lyophilized and stored at −20°C. Furthermore, the chemical profile of TCE was analyzed by using high-pressure liquid chromatograms (HPLC) mass spectrometer as described previously [9]. For subsequent experiments, TCE powder was dissolved in 50% in dimethyl sulfoxide (DMSO) to achieve an indicated concentration with the highest concentration of DMSO less than 0.1%.

### 2.2. Cell Culture and TCE Treatment

HCC (Huh7) cells obtained from Food Industry Research and Development Institute (Hsinchu, Taiwan) was cultured in Dulbecco's modified Eagle's medium (Life Technologies, Grand Island, NY, USA), 10% fetal bovine serum, 2 mM glutamine, 100 U/mL penicillin, 100 *μ*g/mL streptomycin, and 400 ng/mL hydrocortisone. All cell cultures were maintained at 37°C n a humidified atmosphere of 5% CO_2_. For TCE treatment, appropriate amounts of stock solution of TCE were added into culture medium to achieve the indicated concentrations and then incubated with cells for indicated time periods, whereas dimethyl sulfoxide solution without TCE was used as blank reagent.

### 2.3. Determination of Cell Viability (MTT Assay)

For cell viability experiment, a microculture tetrazolium (3-(4,5-dimethylthiazol-2-yl)-2,5-diphenyltetrazolium bromide) colorimetric assay was performed to determine the cytotoxicity of TCE [10, 11]. Huh7 cells were seeded in 24-well plates at a density of 5 × 10^4^ cells/well and treated with TCE at a concentration between 0–100 *μ*g/mL at 37°C for 24 h. After the exposure period, the media was removed, and cells were washed with phosphate-buffered saline (PBS) and then incubated with 20 *μ*L MTT (5 mg/mL) (Sigma chemical Co., St. Louis, MO, USA) for 4 h. The viable cell number per dish is directly proportional to the production of formazan, which can be measured spectrophotometrically at 563 nm following solubilization with isopropanol.

### 2.4. *In Vitro* Wound Closure

Huh7 cells (1 × 10^5^ cells/well) were plated in 6-well plates for 24 h, wounded by scratching with a pipette tip, then incubated with DMEM medium containing 0.5% FBS and treated with or without TCE (0, 25, 50, 75, and 100 *μ*g/mL) for 0, 12, and 24 h. Cells were photographed using a phase-contrast microscope (×100).

### 2.5. Cell Invasion and Migration Assays

Cell invasion and migration were assayed according to the methods described by Yang et al. [12]. After a treatment with TCE (0, 25, 50, 75, and 100 *μ*g/mL) for 24 h, surviving cells were harvested and seeded to Boyden chamber (Neuro Probe, Cabin John, MD, USA) at 10^4^ cells/well in serum-free medium and then incubated for 24 hours at 37°C. For invasion assay, 10 *μ*L Matrigel (25 mg/50 mL; BD Biosciences, MA, USA) was applied to 8 *μ*m pore size polycarbonate membrane filters and the bottom chamber contained standard medium. Filters were then air-dried for 5 h in a laminar flow hood. The invaded cells were fixed with 100% methanol and stained with 5% Giemsa. Cell numbers were counted under a light microscope. The migration assay was carried out as described in the invasion assay with no coating of Matrigel [13].

### 2.6. Determination of MMP-9 by Zymography

The activities of MMP-9 in conditional medium were measured by gelatin zymography protease assays as previously described [12]. Briefly, collected media of an appropriate volume (adjusted by vital cell number) were prepared with SDS sample buffer without boiling or reduction and subjected to 0.1% gelatin-8% SDS-PAGE electrophoresis. After electrophoresis, gels were washed with 2.5% Triton X-100 and then incubated in reaction buffer (40 mM Tris-HCl, pH 8.0; 10 mM CaCl_2_ and 0.01% NaN_3_) for 12 h at 37°C. Then gel was stained with Coomassie brilliant blue R-250.

### 2.7. RNA Preparation and TaqMan Quantitative Real-Time PCR

Total RNA was isolated from Huh7 cells using Trizol (Life Technologies, Grand Island, NY, USA) according to the manufacturer's instructions as previously described [14]. Quantitative real-time PCR analysis was carried out using TaqMan one-step PCR Master Mix (Applied Biosystems). 100 ng of total cDNA was added per 25 *μ*L reactions with MMP-9 or GAPDH primers and TaqMan probes. The MMP-9 and GAPDH primers and probes were designed using commercial software (ABI PRISM Sequence Detection System; Applied Biosystems). Quantitative real-time PCR assays were carried out in triplicate on a StepOnePlus sequence detection system. The threshold was set above the nontemplate control background and within the linear phase of target gene amplification to calculate the cycle number at which the transcript was detected.

### 2.8. Preparation of Total Cell Lysates and Nuclear Fraction

For total cell lysates preparation, cells were rinsed with PBS twice and scraped with 0.2 mL of cold RIPA buffer containing protease inhibitors cocktail, and then vortexed at 4°C for 10 min. Cell lysates were subjected to a centrifugation of 10,000 rpm for 10 min at 4°C, and the insoluble pellet was discarded. Nuclear extracts were obtained using a modification of a previously described method [13]. Briefly, harvested cells were scraped and lysed with buffer A (10 mM HEPES, 10 mM KC1, 0.1 mM EDTA, 1.5 mM MgCl_2_, 0.2% NP40, 1 mM DTT, and 0.5 mM phenylmethylsulfonyl fluoride), followed by vortexing to shear the cytoplasmic membranes and nuclear pellets were collected by a centrifugation at 3000 rpm for 30 s at 4°C. Nuclear proteins were extracted with high-salt buffer B (20 mM HEPES, 25% glycerol, 1.5 mM MgCl_2_, 0.1 mM EDTA, 420 mM NaCl, 1 mM DTT, and 0.5 mM phenylmethylsulfonyl fluoride). The protein concentration of total cell lysates and nuclear fraction were determined by Bradford assay [15].

### 2.9. Western Blot Analysis

The cell lysates or nuclear extracts were separated in a 10% polyacrylamide gel and transferred onto a nitrocellulose membrane. The blot was subsequently incubated with 5% nonfat milk in Tris-buffered saline (20 mM Tris, 137 mM NaCl, pH 7.6) for 1 h to block non-specific binding and then overnight with polyclonal antibodies against MMP-9, TIMP-1, NF-*κ*B, I*κ*B, c-Jun, and c-Fos. Blots were then incubated with a horseradish peroxidase goat anti-rabbit or anti-mouse IgG for 1 h. Afterwards, signal was detected by using enhanced chemiluminescence (ECL) commercial kit (Amersham Biosciences) and relative photographic density was quantitated by scanning the photographic negatives on a gel documentation and analysis system (AlphaImager 2000, Alpha Innotech Corporation, San Leandro, CA, USA).

### 2.10. Transfection and MMP-9 Promoter-Driven Luciferase Assays

Huh7 cells were seeded at a concentration of 5 × 10^4^ cells per well in 6-well cell culture plates. After 24 h of incubation, pGL3-basic (vector) and MMP-9 promoter plasmid were cotransfected with a *β*-galactosidase expression vector (pCH110) into cells using Turbofect (Fermentas, Carlsbad, CA). After 12 h of transfection, cells were treated with vehicle or TCE (0, 25, 50, 75, and 100 *μ*g/mL) for 24 h. The cell lysates were harvested, and luciferase activity was determined using a luciferase assay kit. The value of the luciferase activity was normalized to transfection efficiency and monitored by *β*-galactosidase expression.

### 2.11. Chromatin Immunoprecipitation Analysis (ChIP)

Chromatin immunoprecipitation analysis was performed as described previously [14]. DNA immunoprecipitated with anti-NF-*κ*B and anti-AP-1 was purified and extracted using phenol-chloroform. Immunoprecipitated DNA was analyzed by PCR or quantitative PCR by using specific primers.

### 2.12. Statistical Analysis

Statistical significances of difference throughout this study were calculated by Student's *t*-test (Sigma-Stat 2.0, Jandel Scientific, San Rafael, CA, USA). A difference at *P* < 0.05 was considered to be statistically significant and the experiments were repeated three times.

## 3. Results

### 3.1. Effects of TCE on Huh7 Cell Viability

**Figure 1:**
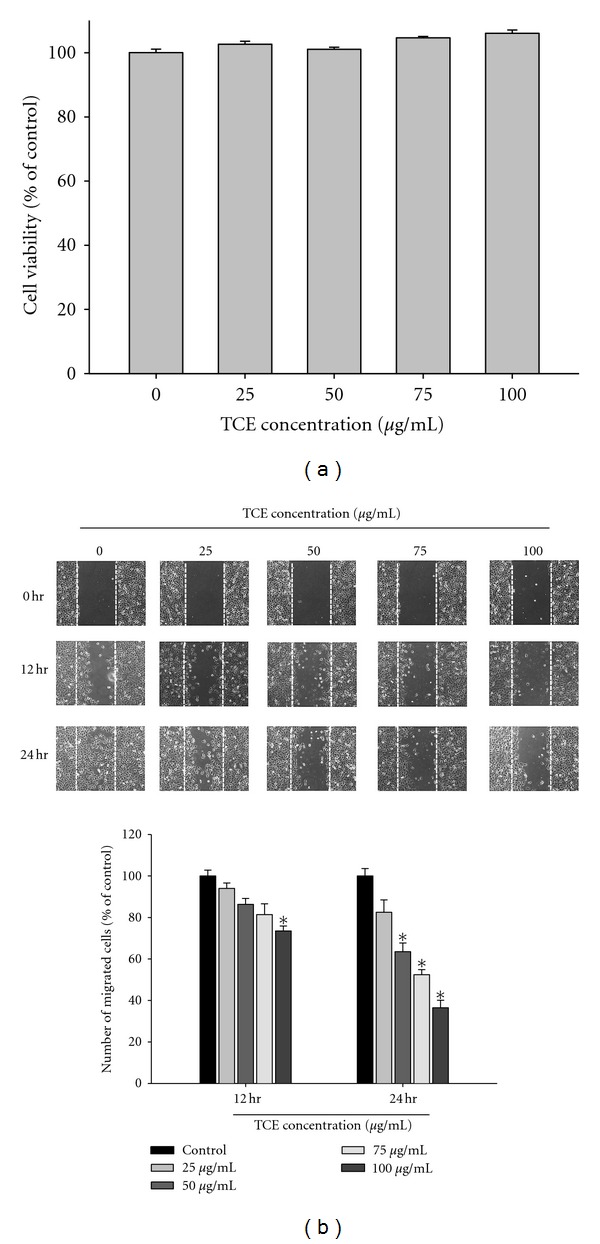
Effect of TCE on cell viability and *in vitro* wound closure in Huh7 cells. (a) Huh7 cells were treated with TCE (0, 25, 50, 75 and 100 *μ*g/mL) for 24 h before being subjected to a MTT assay for cell viability. The values represented the means ± SD of at least three independent experiments. (b) Huh7 cells were wounded and then treated with vehicle (DMSO) or TCE (0, 25, 50, 75 and 100 *μ*g/mL) for 0 h, 12 h and 24 h in 0.5% FBS-containing medium. At 0, 12, and 24 h, phase-contrast pictures of the wounds at three different locations were taken.

Figure 1(a) displays the cytotoxic effects of various concentrations of TCE (0 *μ*g/mL to 100 *μ*g/mL) on Huh7 cells. Results from MTT assay revealed that TCE did not influence Huh7 cell viability at all tested concentrations. A lower TCE concentration range was, thus, used for all subsequent experiments.

### 3.2. Effects of TCE on Wound Closure, Invasion, and Migration of Huh7 Cells *In Vitro*

**Figure 2:**
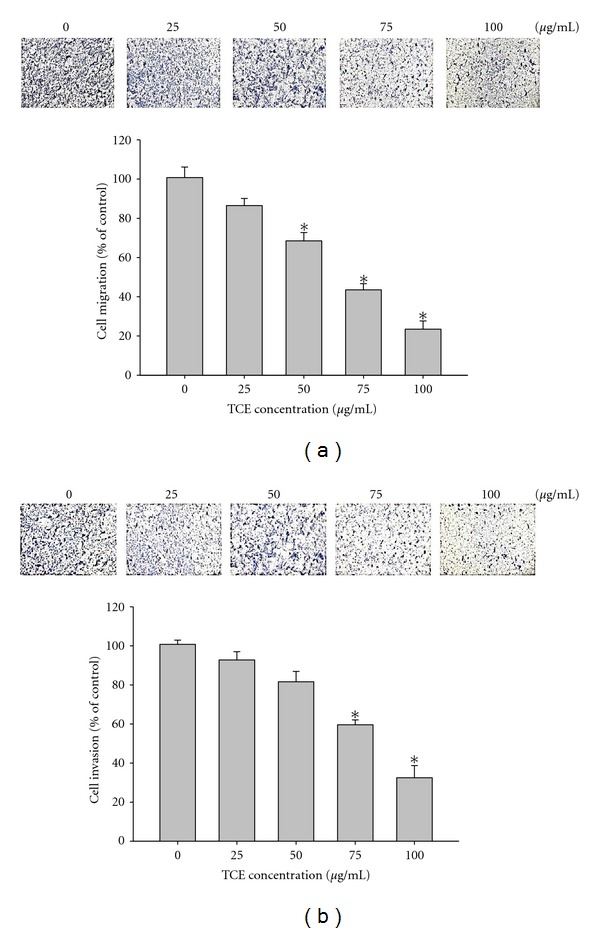
Effect of TCE on cell migration and invasion in Huh7 cells. (a) The cell migration and (b) cell invasion were measured using a Boyden chamber for 16 h and 24 h with polycarbonate filters, respectively. The migration and invasion abilities of Huh7 cells were quantified by counting the number of cells that invaded the underside of the porous polycarbonate as described in Section 2. The values represented the means ± SD of at least three independent experiments. *P < 0.05 as compared with the vehicle group.

Figure 1(b) displays the findings from wound closure assay to evaluate the effects of TCE on Huh7 cell migration, showing representative photographs of Huh7 cells migrating into scratch wounds following treatment with TCE. Figures 2(a) and 2(b) demonstrate the effects of TCE on cell migration and invasion in Huh7 cells treated with 0, 25, 50, 75, and 100 *μ*g/mL TCE for 16 h (cell migration) and 24 h (cell invasion). Cell migration and invasion assays using a Boyden chamber revealed that TCE induced marked reductions in the invasion and migration abilities of Huh7 cells in a concentration-dependent manner.

### 3.3. Effects of TCE on the Expression of MMP-9 and Its Endogenous Inhibitor

**Figure 3:**
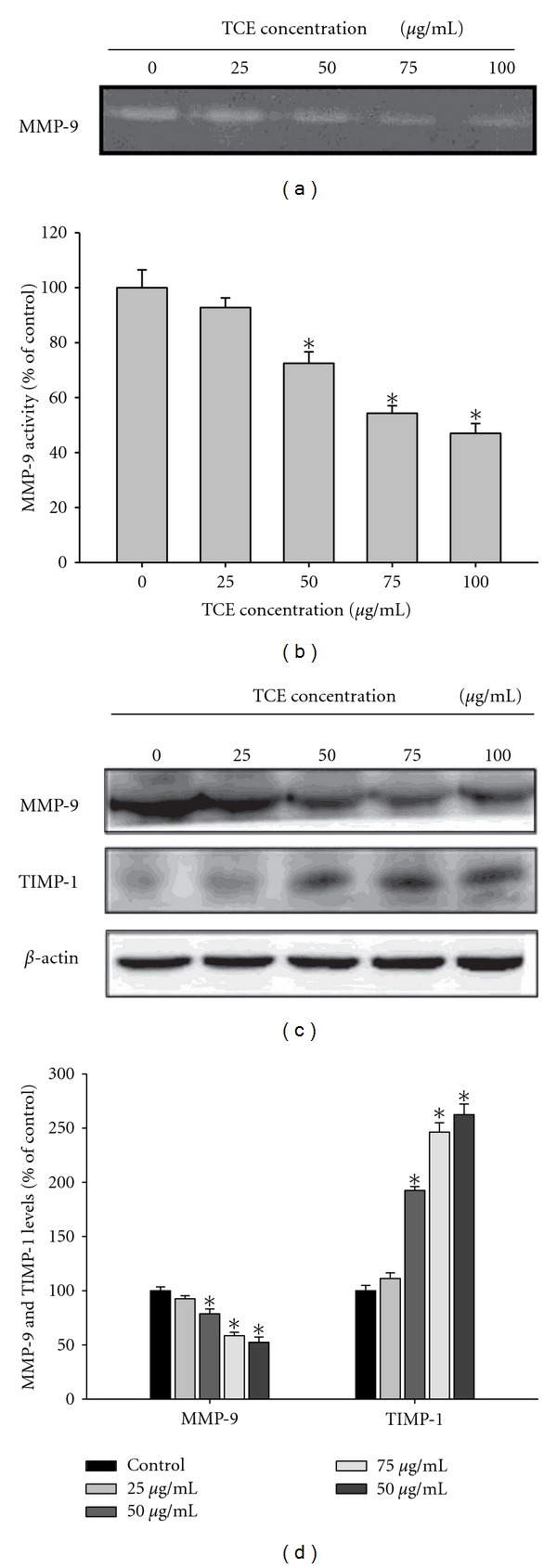
Effects of TCE on the activity and protein level of MMP-9 and the protein level of the endogenous inhibitor TIMP-1. (a) Huh7 cells were treated with TCE (0, 25, 50, 75 and 100 *μ*g/mL) for 24 h and then subjected to gelatin zymography to analyze the activity of MMP-9. (b) Huh7 cells were treated with TCE (0, 25, 50, 75 and 100 *μ*g/mL) for 24 h and then subjected to western blotting to analyze the protein levels of MMP-9 and TIMP-1. Quantitative results of MMP-9 and TIMP-1 protein levels which were adjusted with *β*-actin protein level. The values represented the means ± SD of at least three independent experiments. *P < 0.05 as compared with the vehicle group.

The Huh7 cells were treated with TCE (0, 25, 50, 75, and 100 *μ*g\mL) for 24 h, and then subjected to gelatin zymography to analyze MMP-9 activity. As shown in Figures 3(a)-3(b), TCE treatment reduced MMP-9 activity in a dose-dependent manner.  Figure 3(c) shows Western blot analysis of the expression of MMP-9 and its endogenous inhibitor TIMP-1. The MMP-9 and TIMP-1 protein levels were adjusted using *β*-actin. Following TCE treatment, MMP-9 expression decreased significantly, whereas TIMP-1 expression increased (Figure 3(d)).

### 3.4. Effects of TCE on MMP-9 Transcription

**Figure 4:**
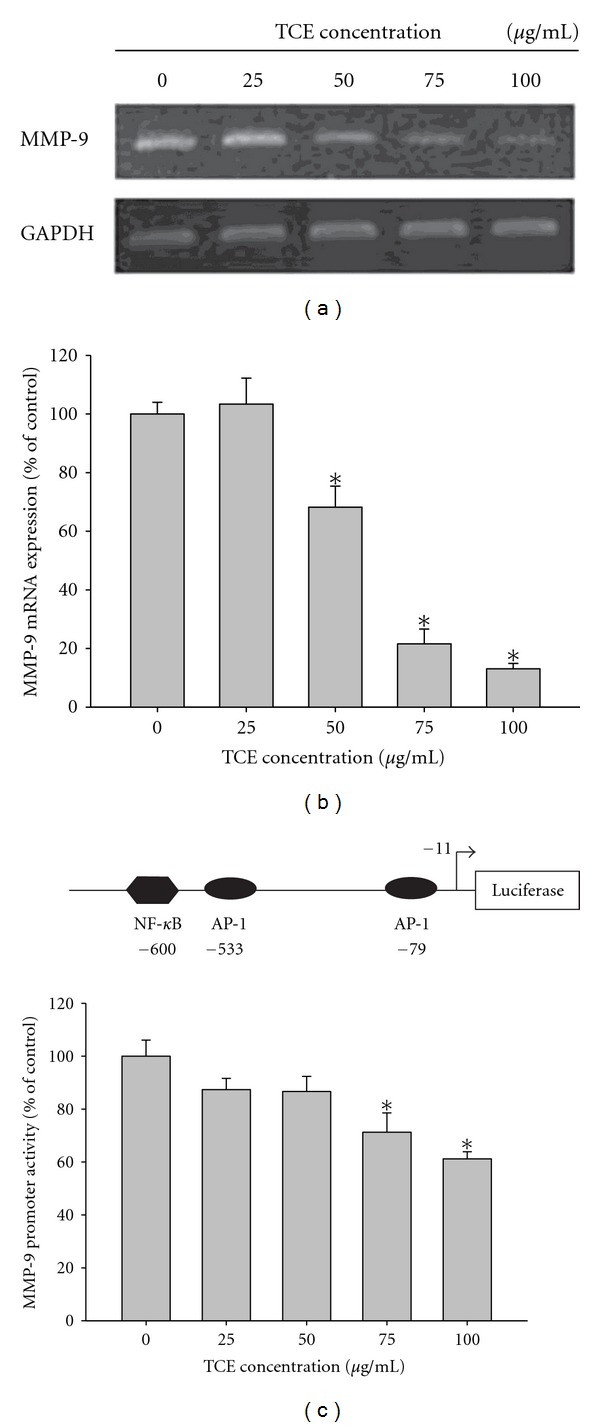
TCE suppresses MMP-9 expression at a transcriptional level. Huh7 cells were treated with TCE (0, 25, 50, 75, and 100 *μ*g/mL) for 24 h and then subjected to (a) reverse transcription-PCR and (b) quantitative real-time PCR to analyze the mRNA expression of MMP-9. (c) MMP-9 promoter reporter assay to analyze the promoter activity of MMP-9. Luciferase activity, determined in triplicates, was normalized to *β*-galactosidase activity. The values represented the means ± SD of at least three independent experiments. *P < 0.05 as compared with the vehicle group.

To evaluate the inhibitory effects of TCE on MMP-9 expression in Huh7 cells, Huh7 cells were treated with 0, 25, 50, 75, and 100 *μ*g/mL TCE for 24 h, and then subjected to reverse transcriptase polymerase chain reaction (RT-PCR) and real-time PCR to analyze mRNA levels. After treatment with various concentrations of TCE, the levels of MMP-9 mRNA showed marked decreases in a concentration-dependent manner (Figures 4(a) and 4(b)). Promoter analysis using a luciferase assay kit identified significant inhibition of the luciferase activities of MMP-9 (Figure 4(c)). These results indicate that TCE regulates the expression of MMP-9, at least partially, at a transcriptional level.

### 3.5. The Transcription Factors NF-*κ*B and AP-1 Are the Key Regulators Involved in Transcriptional Inhibition of MMP-9 by TCE

**Figure 5:**
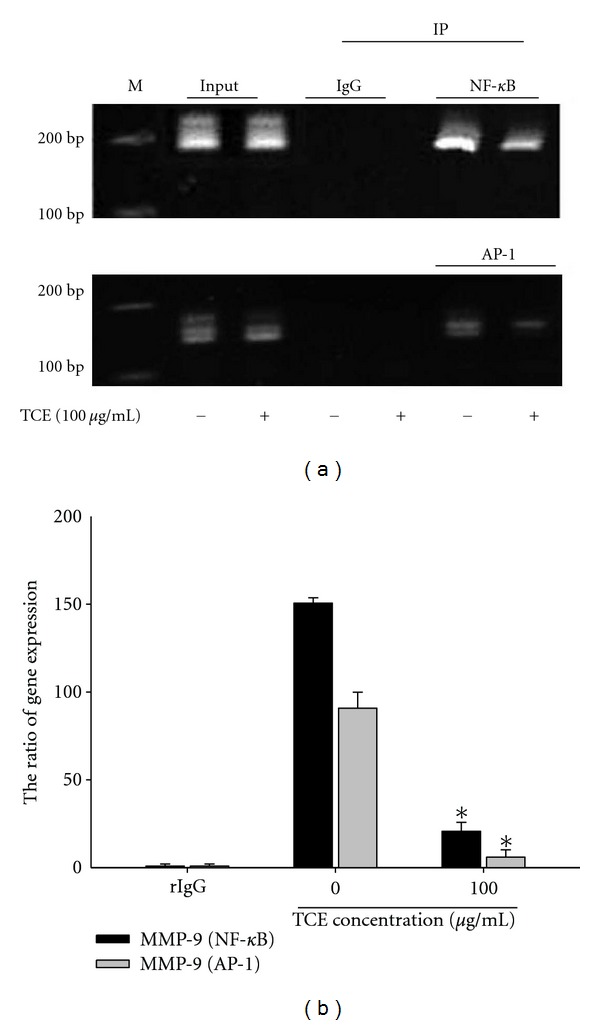
Critical role of AP-1 and NF-*κ*B in TCE-induced transcriptional inhibition of MMP-9 in Huh7 cells. Huh7 cells were treated with TCE (0, 100 *μ*g/mL) for 24 h and then the nuclear fraction was prepared as described in Section 2. ((a) and (b)) Levels of AP-1 and NF-*κ*B in the nucleus were immunodetected with AP-1- and NF-*κ*B-, specific antibodies, respectively.

Sequence analysis of the MMP-9 promoter revealed a number of cis-acting regulatory elements that could potentially be involved in the regulation of MMP-9 expression, including NF-*κ*B and AP-1. Chromatin immunoprecipitation (ChIP) assay then investigated the involvement of these transcription factors in the TCE-induced transcriptional inhibition of MMP-9 (Figure 5(a)). Results from quantitative real-time PCR indicated that TCE significantly reduced the binding of NF-*κ*B and AP-1 to the MMP-9 promoters (Figure 5(b)).

**Figure 6:**
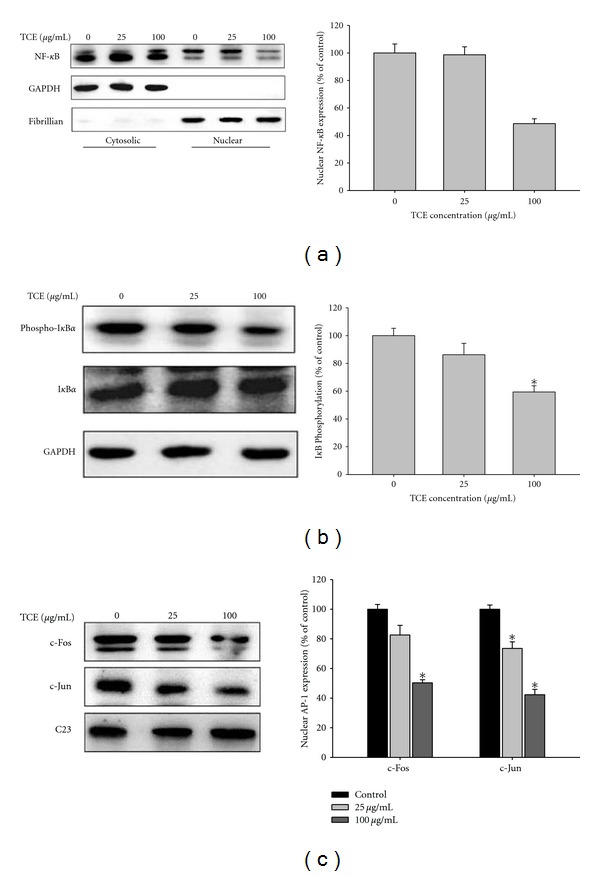
NF-*κ*B and AP-1 expressions in TCE-induced transcriptional inhibition of Huh7 cells. Huh7 cells were treated with TCE 25 and 100 *μ*g/mL for 24 h and then the nuclear fraction was prepared as described in Section 2. Levels of NF-*κ*B (a), I*κ*B (b), c-fos, and c-Jun (c) in the nucleus were immunodetected with NF-*κ*B-, I*κ*B-, c-fos-, and c-Jun-specific antibodies, respectively. Representative results of NF-*κ*B, I*κ*B, c-fos, and c-Jun protein levels determined by western blot analysis. The values represented the means ± SD of at least three independent experiments. *P < 0.05 as compared with the vehicle group.

To further investigate the involvement of AP-1 and NF-*κ*B in the transcriptional regulation of MMP-9 by TCE in Huh7 cells, the effects of TCE on nuclear translocation of NF-*κ*B, c-fos, and c-Jun were evaluated. Treatment of Huh7 cells with 0, 25, and 100 *μ*g/mL TCE reduced nuclear translocation of NF-*κ*B, and phosphorylation of I*κ*B, c-Fos, and c-Jun (Figures 6(a) to 6(c)). These findings indicated that TCE might induce transcriptional inhibition of MMP-9 in Huh7 cells by suppressing NF-*κ*B and AP-1 nuclear translocation and MMP-9 promoter binding activity.

## 4. Discussion

Herbal products are used worldwide in the prevention and treatment of various chronic diseases, and their potential anticancer and antimetastatic effects are currently under investigation. *Terminalia catappa* leaf extracts exert a range of biological effects on cells, including antioxidant and hepatoprotective activity on hepatocytes and liver mitochondria, and preventive activity against hepatocyte apoptosis [16–19]. In previous studies, water extracts of TCE could effectively reduce carbon-tetrachloride- (CCl_4_-) induced hepatotoxicity and bleomycin-induced genotoxicity of Chinese hamster ovary cells [20, 21]. Tang et al. reported that ethanol extracts of TCE contain higher active components than water extracts of TCE [8]. In an earlier study, the same group identified that chloroform extracts of TCE induce hepatoprotective effects that might be related to the regulation of liver IL-6 gene expression [22]. Different methods of extraction of TCE can, therefore, influence the physiological nature of the active compound and modify its effects directly.

As described in previous studies, the protective activities of TCE against liver mitochondrial damage induced by carbon tetrachloride (CCl_4_) might be related to the inhibition of IL-6 gene overexpression [20, 23]. Studies have also suggested that its protective effects might be related to the scavenging of reactive oxygen species (ROS) [20, 21, 24]. The groups of Fan and Tang speculated that TCE might prevent DNA damage and carcinogenesis by inhibiting ROS generation or reducing DNA adduct formation [19, 25]. In the study of Wen et al., *T. catappa* L. hydrophilic extract (TCLW) inhibited MMP-1, MMP-3, and MMP-9 expressions at a range of concentrations and promoted the expression of type I procollagen by inhibiting MMP-1, -3, and -9 activities. This afforded it antiaging activity and potential cosmetic use [24]. Ko et al. also described that SC-CO_2_ extract of TCE exhibited potent antimutagenicity and demonstrated higher cytotoxicity against HCC cells than against normal liver cells. Therefore, TCE is a potent antioxidant and might provide a novel source of biomedicinal phytochemicals for cancer prevention [26]. However, constraints on the use of herbal products for prevention of cancer metastasis have yet to be cleared, and the underlying biological mechanisms of their activities have yet to be fully characterized. Our group's previous studies identified that TCE induces antimetastatic effects in lung cancer (A549, Lewis lung carcinoma, LLC) and oral cancer cells (SCC-4) [5, 9]. The present study's results indicate that TCE also exerts inhibitory effects on HCC cell metastasis through regulation of MMP-9. Morioka et al. reported that TCE has a potent short-term chemopreventive effect on biomarkers of colon carcinogenesis and might be associated with inhibition of the development of aberrant crypt foci (ACF) and beta-catenin-accumulated crypts (BCACs) [27]. It is, therefore, possible that other proteases are involved in the antimetastatic effects of TCE on HCC. Further investigation is needed to confirm the involvement of other antimetastatic mechanisms, and changes in the expression of proteases, in TCE-induced effects. 

Transcription of the MMP-9 gene is regulated by upstream sequences, including motifs corresponding to NF-*κ*B or AP-1 binding sites [6, 28, 29]. In the present study, TCE inhibited the binding of NF-*κ*B and AP-1 to the MMP-9 promoter in Huh7 cells. The transcription nuclear factors NF-*κ*B and AP-1 can promote tumorigenesis and are linked to invasion and metastasis. In the study by Aggarwal, inhibition of NF-*κ*B and AP-1 activities was effective in the prevention and treatment of cancer [30]. This result was similar to the group's previous finding that norcantharidin (NCTD) exerted inhibitory effects on metastasis in HCC cells [6]. Activation of NF-*κ*B occurs through the phosphorylation of I*κ*B*α*. This releases NF-*κ*B, which then translocates from the cytosol to the nucleus to regulate gene expression at a transcriptional level. In the present study, TCE treatment resulted in inhibition of NF-*κ*B DNA binding activity, accompanied by inhibition of I*κ*B*α* phosphorylation, leading to the downregulation of MMP-9. 

**Figure 7:**
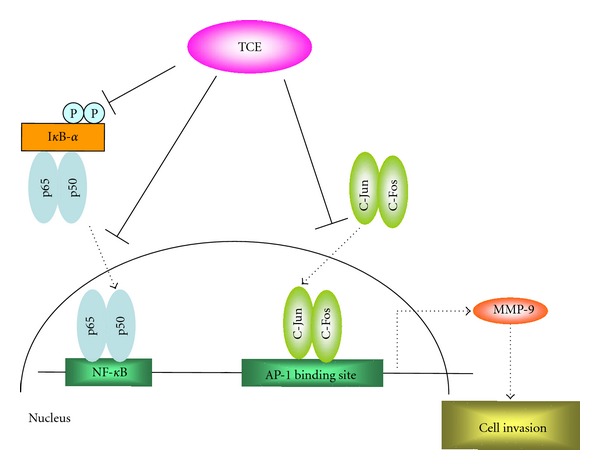
Proposed signal transduction pathways by which TCE inhibits invasion of Huh7 cells.

In conclusion, the present study's findings demonstrate that TCE exerts inhibitory effects on several critical stages of metastasis, including cell invasion and migration, by regulating the activities of metastasis-associated proteases and their natural inhibitors. Terminalia Catappa leaf extracts might, therefore, have potential use in the development of preventive and treatment agents for cancer metastasis. *Terminalia catappa* leaf extracts inhibit NF-*κ*B and AP-1 DNA binding activities effectively, resulting in the downregulation of MMP-9 expression and the inhibition of metastasis (Figure 7). By targeting the signal transduction mediators and transcriptional factors involved in the TCE antimetastatic process in human hepatoma cells, it might be possible to develop specific mediators to inhibit cell metastasis. Further *in vivo* analysis is needed to confirm the effectiveness of TCE in the prevention or treatment of hepatoma invasion or migration.

## Abbreviations

TCE: *Terminalia catappa* leaf extracts
ECM: Extracellular matrix
MMP: Matrix metalloproteinase
TIMP: Tissue inhibitor of metalloproteinase
HCC: Hepatocellular carcinoma
ChIP: Chromatin immunoprecipitation.
