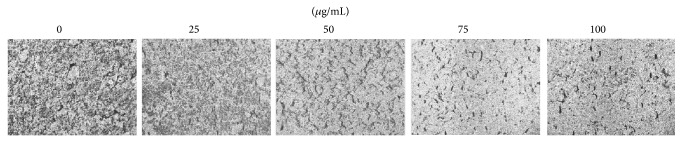# Corrigendum to “*Terminalia catappa* Exerts Antimetastatic Effects on Hepatocellular Carcinoma through Transcriptional Inhibition of Matrix Metalloproteinase-9 by Modulating NF-*κ*B and AP-1 Activity”

**DOI:** 10.1155/2015/326234

**Published:** 2015-11-24

**Authors:** Chao-Bin Yeh, Ming-Ju Hsieh, Yih-Shou Hsieh, Ming-Hsien Chien, Pen-Yuan Lin, Hui-Ling Chiou, Shun-Fa Yang

**Affiliations:** ^1^School of Medicine, Chung Shan Medical University, 110 Chien-Kuo N. Road, Section 1, Taichung 402, Taiwan; ^2^Department of Emergency Medicine, Chung Shan Medical University, 110 Chien-Kuo N. Road, Section 1, Taichung 402, Taiwan; ^3^Department of Emergency Medicine, Chung Shan Medical University Hospital, 110 Chien-Kuo N. Road, Section 1, Taichung 402, Taiwan; ^4^School of Medical Laboratory and Biotechnology, Chung Shan Medical University, 110 Chien-Kuo N. Road, Section 1, Taichung 402, Taiwan; ^5^Institute of Biochemistry and Biotechnology, Chung Shan Medical University, 110 Chien-Kuo N. Road, Section 1, Taichung 402, Taiwan; ^6^Wan Fang Hospital, Taipei Medical University, Taipei, Taiwan; ^7^Graduate Institute of Clinical Medicine, College of Medicine, Taipei Medical University, Taipei, Taiwan; ^8^School of Pharmacy, Taipei Medical University, Taipei, Taiwan; ^9^Institute of Medicine, Chung Shan Medical University, 110 Chien-Kuo N. Road, Section 1, Taichung 402, Taiwan; ^10^Department of Medical Research, Chung Shan Medical University Hospital, 110 Chien-Kuo N. Road, Section 1, Taichung 402, Taiwan

In the paper “*Terminalia catappa* Exerts Antimetastatic Effects on Hepatocellular Carcinoma through Transcriptional Inhibition of Matrix Metalloproteinase-9 by Modulating NF-*κ*B and AP-1 Activity” [1], there is a misplaced figure in Figures 1(b) and 2(b) and the corrected version of Figures 1(b) and 2(b) is herein provided. The correction does not affect the findings or conclusion of the study.

## Figures and Tables

**Figure 1:**
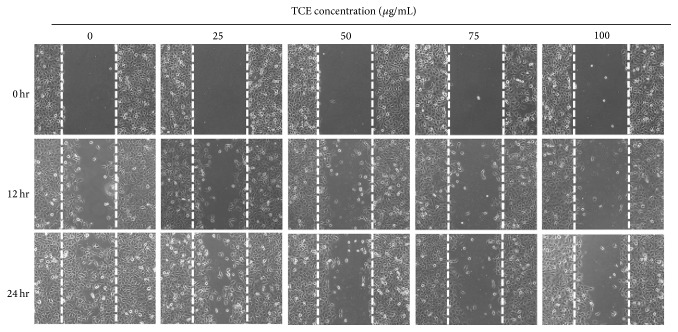


**Figure 2:**